# The Effect of a Video-Based Game Exercise Program on Motor Skills, Proprioception, and Cognitive Functions in Individuals With Intellectual Disabilities

**DOI:** 10.1155/oti/8410494

**Published:** 2025-01-30

**Authors:** Elif Diril, Burak Menek, Ahmet Emir, Devrim Tarakci, Ela Tarakci

**Affiliations:** ^1^Department of Occupational Therapy, Institute of Health Sciences, Istanbul Medipol University, Istanbul, Turkey; ^2^Department of Physiotherapy and Rehabilitation, Faculty of Health Sciences, Istanbul Medipol University, Istanbul, Turkey; ^3^Department of Occupational Therapy, Faculty of Health Sciences, Istanbul Medipol University, Istanbul, Turkey; ^4^Department of Physiotherapy and Rehabilitation, Faculty of Health Sciences, Istanbul University-Cerrahpasa, Istanbul, Turkey

**Keywords:** exercise, exergame, intellectual disabilities, MOXO-continuous performance test, proprioception

## Abstract

**Background:** Individuals with intellectual disability (ID) exhibit various problems, such as attention, learning, and physical–motor difficulties. The study is aimed at investigating the effects of video game–based therapy programs on cognitive and physical functions in individuals with ID.

**Methods:** The study, conducted through a three-arm randomized trial, involved 45 adults aged 18–30 with mild to moderate ID. Participants were divided into three groups: Group 1 received home-based video game–based therapy, supervised video game–based therapy, and occupational therapy–based activity training. Group 2 received video game–based therapy supervised by a therapist and an occupational therapy–based activity training program. Group 3 received occupational therapy–based activity training only. MOXO d-CPT was used to evaluate attention; sit-to-stand test, joint position sense, and nine-hole peg test were used to assess physical–motor functionality.

**Results:** Results showed significant improvements in attention, functional performance, proprioception, and fine motor skills in all groups (*p* < 0.05). Group 1 was superior to Group 3 in nondominant JPS-flexion, sit-to-stand test, and nondominant JPS-abduction parameters (*p* < 0.017). When comparing Group 1 and Group 2, Group 1 was found to be more effective in the sit-to-stand test parameter compared to Group 2 (*p* < 0.017), while the other parameters of the two groups were similar to each other (*p* > 0.017). There were no significant differences between groups for different outcome measures (*p* > 0.017).

**Conclusion:** The study suggests that video-based game exercises combined with occupational therapy interventions can effectively enhance cognitive functions, functional performance, proprioception, and fine motor skills in adults with ID.

**Trial Registration:** ClinicalTrials.gov identifier: NCT06097819

## 1. Introduction

Intellectual disabilities (IDs) are identified by their emergence during development, encompassing cognitive impairments (such as reasoning, learning, and problem-solving) and deficiencies in adaptive behavior across conceptual, social, and practical spheres. These deficits hinder functionality in various aspects of daily life [1]. Notable constraints in cognitive abilities and adaptive skills characterize ID. The evaluation of ID traditionally is done through psychometric tests of intelligence, known as an intelligence quotient (IQ) test, which currently includes evaluation of adaptive functioning or life skills. Individuals with IDs frequently encounter difficulties in attention, motor function, and awareness [2]. In individuals with ID, many motor disorders can be observed that stem from a lack of integration among the visual, vestibular, and proprioceptive systems [3, 4].

Occupational therapy interventions assess individuals with ID to identify challenges and provide meaningful and purposeful activities to promote independence despite cognitive limitations. These interventions adopt a person-centered approach to enhance participation in daily life activities such as school, work, nutrition, and sleep. Environmental interventions may also be needed to support the individual in fully participating. These interventions play a crucial role in rehabilitation programs that foster the development of individuals through occupations and activities [5].

Karhula, Heiskanen, and Salminen have reported that occupational therapy interventions can enhance the participation of individuals with ID across daily activities, emphasizing the importance of occupational therapy for individuals with ID to increase involvement in school and home environments. For instance, occupational therapy interventions have been shown to help a child with ID participate more fully in classroom activities by improving fine motor skills, leading to better handwriting and greater independence in school tasks. Similarly, in the home environment, such interventions can enable individuals to take on more daily responsibilities, such as self-care routines. These examples underscore the critical role of occupational therapy in promoting meaningful engagement in school and home settings, highlighting the need for continued focus on these practices in future research and clinical applications [6].

Adults with ID have long faced occupational challenges and can benefit from the therapeutic and functional use of assistive technology and other technological supports [7]. Technologies used to support the participation of adults with ID include common applications of information and communication technologies, computerization, artificial intelligence, and virtual reality (VR); these technologies can help in daily life activities, community living, and social inclusion [8–10].

Technological advancements have led to the development of specific exercise protocols, including video game–based therapies, which are increasingly utilized in the field. Such technological rehabilitation methods are categorized under complementary approaches for individuals requiring rehabilitative care [11]. The development of motion-capturing sensors plays a crucial role in these rehabilitation processes using camera systems, thus eliminating the need for additional equipment. Devices such as Microsoft Kinect and Nintendo Wii are examples of technologies used in this context. Furthermore, exercise programs incorporating video games are designed as a therapeutic tool, merging gameplay with physical exercises to enhance the individual's physical activity and functionality [12]. It is suggested that active video games can be a promising supportive treatment method for individuals with ID [13].

Taylor et al. have reported the significance of developing motion-sensor games for adults with ID. They noted that motion-sensor games are motivating, beneficial, and effective in encouraging physical exercise among adults with ID [14]. Another study reported that video game–based therapy may be a beneficial technological approach to promote physical activity in individuals with ID. Additionally, it has been emphasized that the literature on technology-based interventions in individuals with ID is limited and that there is a need for further research in this area [15]. Another systematic review highlighted that technology-based interventions have shown the potential to improve physical activity in individuals with ID. However, it emphasized that the limited number of studies in the literature, often conducted with small sample sizes, underscores the need for more comprehensive research on technology-based interventions for individuals with ID in future studies [16]. A study by Kwon, Maeng, and Chung reported that a technology-based exergame program designed for individuals with developmental disabilities positively impacted fundamental motor skills and physical fitness. However, they emphasized the need for more comprehensive studies in the future to further investigate the effectiveness of technology-based interventions in this population [17].

Our hypothesis for this study was that combined video game–based and occupational therapy interventions could positively affect motor skills, cognitive functions, and proprioception in individuals with ID. The study is aimed at investigating the effects of these combined interventions, specifically the video game–based therapy programs integrated with occupational therapy, on proprioception, motor skills, and cognitive functions in individuals diagnosed with ID.

## 2. Methods

### 2.1. Study Design

A three-arm randomized trial study was conducted between September 2023 and February 2024. The study was conducted in Bağcılar Municipality Community-Based Care Center in Bağcılar Region of Istanbul, Turkey. Ethical approval was approved by Istanbul Medipol University Ethical Committee with Number E-10840098-772.02-212. Each group received one of three distinct intervention programs, which will be described subsequently. All participants received assessments before and after the intervention programs.

### 2.2. Participants

**Figure 1:**
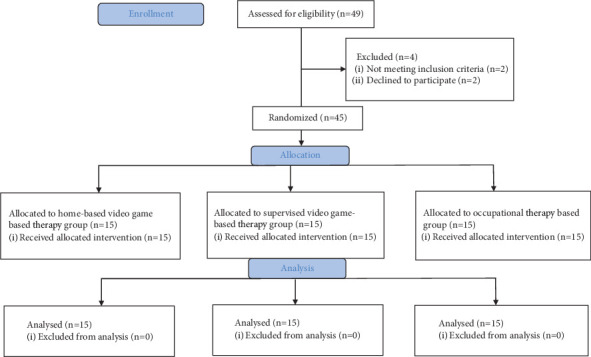
Design and flow of participants through the trial.

The study included 45 adults with mild to moderate IDs aged 18–30 (Figure 1). All participants and their legal guardians volunteered and signed the informed consent to participate in the study. Inclusion criteria were adults aged between 18 and 30 with mild to moderate ID according to the Diagnostic and Statistical Manual of Mental Disorders–V (DSM-V) [1] and able to understand and cooperate with video games. Exclusion criteria were having behavioral problems, inability to follow instructions for the games, being sensitive to light, and visual problems. All participants received eligibility screening before the study [1].

### 2.3. Intervention Programs

The numbers from 1 to 45 were randomly divided into three groups (Group 1, Group 2, and Group 3) using the website randomizer.org. Forty-five individuals were included in the study and were randomized into these three groups. The numbers from 1 to 45 were written on paper and placed in a closed box. Participants were asked to draw a number from this box randomly. Participants were assigned to the corresponding group based on the numbers they drew and the group assignment determined by randomizer.org. One of the therapists, who was not involved in the primary research, was responsible for conducting the evaluation and the treatment procedures. This approach ensured that the participants were unaware of the specific treatment group they were assigned, maintaining the integrity of the single-blind design. By having a separate therapist handle the evaluations and treatments, the principal investigators could remain blinded to the participants' group assignments, thereby reducing the potential for bias and enhancing the reliability of the study's findings. Each group received a 6-week intervention program. Group 1 (*n* = 15) received a home-based video game–based therapy program, supervised video game–based therapy, and an occupational therapy–based activity training program. Group 2 (*n* = 15) received video game–based therapy supervised by a therapist in addition to an occupational therapy–based activity training program. Group 3 (*n* = 15) received an occupational therapy–based activity training program only.

#### 2.3.1. Home-Based Video Game–Based Therapy Program

Participants in home-based video game–based therapy received active video game–based therapy programs through mobile devices. The games were custom games developed for physical activity and rehabilitation (Becure Gmbh). Video games were played on a tablet at home five times per week for 6 weeks. Each video gaming session lasted 10 min (Table S1).

#### 2.3.2. Supervised Video Game–Based Therapy Program

Custom video games using Leap Motion, Kinect V2, and Wii Balance Board (WBB) sensors were applied two times per week for 6 weeks. Each session lasted 30 min under the supervision of the occupational therapist (Table S2).

#### 2.3.3. Occupational Therapy–Based Activity Training Program

All groups received an occupational therapy–based activity training program. All activities included weight bearing, balance, fine motor tasks, and cognitive tasks. The activity training program was applied two times a week for 6 weeks. Each session lasted 30 min under the supervision of the occupational therapist (Table S3).

### 2.4. Outcome Measures

Participants were evaluated at baseline and following intervention. The demographic characteristics of the participants were recorded via a demographic data questionnaire. Attention and reaction time were evaluated by MOXO d-CPT. Proprioception was evaluated by Kinect-based analysis system (Becure Extremity Rom). The 30-s sit-to-stand (STS) test was used to evaluate neuromuscular functionality. The nine-hole peg test (NHPT) evaluated fine motor skills.

#### 2.4.1. Primary Outcome Measures

##### 2.4.1.1. Attention and Reaction Time Assessments

MOXO d-CPT was used to assess the attention and reaction time of participants. MOXO d-CPT is a continuous performance test that consists of eight blocks. The test is conducted by a computer and does not require any special equipment. The test includes 53 trials in each block. In each trial, there are targets and nontargets. Participants are asked to press the spacebar if they see the target and avoid pressing the spacebar if they know the nontarget. There are also distracters placed in each trial to assess attention and reaction time. There are four performance indicators for examiners: (a) attention, (b) timeliness, (c) hyperactivity, and (d) impulsivity [18].

##### 2.4.1.2. Fine Motor Skills Assessment

The NHPT was used to assess the fine motor skills of participants. The NHPT is a practical assessment tool to evaluate timed fine motor performance. It consists of a nine-hole platform and nine pegs. During the test, participants were asked to place the pegs into holes and remove them as quickly as possible. Only the tested hand is used during the test, while the other hand stabilizes the board [19].

#### 2.4.2. Secondary Outcome Measures

##### 2.4.2.1. Proprioception Assessments

Proprioception is defined as the perception of joint and body movement and position of the body, or body segments, in space [20]. Different techniques have been proposed to assess proprioception. The main techniques are threshold to detection of passive motion (TTDPM), joint position reproduction, and active movement extent discrimination assessment (AMEDA) [21]. In this study, joint position reproduction tests were used to evaluate shoulder abduction and flexion.

##### 2.4.2.2. Functional Performance

The 30-s STS test was used to assess the functional performance of each participant. In this test, the individual is asked to stand from a chair and immediately sit, continuing this task for 30 s. STS is a reliable, practical, and effective test for lower extremity functionality [22].

### 2.5. Statistical Analysis

The sample size was determined as 12 people for each group using the NHPT effect size (ES): 1.29, mean difference (MD): 22.52, and standard deviation (SD): 5.46 [23] in the G⁣^∗^Power program [24] with a 5% margin of error and 80% power. We planned to recruit 15 people for each group because of the possible dropouts. Intragroup and intergroup analyses of data with normal distribution were performed with “one-way analysis of variance (ANOVA).”

The “Wilcoxon signed-rank” test was used to compare the pre-and posttreatment results of the groups. Difference analysis between groups was performed using the post hoc test “Tukey HSD.” The significance value was accepted as *p* < 0.05 for the one-way ANOVA, and the significance value for the post hoc test was *p* < 0.017.

## 3. Results

### 3.1. Baseline Characteristics

Individuals with a diagnosis of ID were selected among those who applied to the Bağcılar Municipality Community-Based Care Center. Individuals were divided into three groups: Group 1 (*n* = 15), Group 2 (*n* = 15), and Group 3 (*n* = 15). Age and gender data were collected to analyze demographic characteristics at the baseline. There were seven men and eight women in Group 1, eight women and seven men in Group 2, and eight men and seven women in Group 3. The mean age of Group 1 was 22.20 years, the mean age of Group 2 was 22.40 years, and the mean age of Group 3 was 22.93 years. There was no statistical difference between the baseline demographic characteristics of the groups (*p* > 0.05).

### 3.2. Results of Outcome Measures

The comparison of pre-and posttreatment evaluation parameters within each group is presented in Table 1. When comparing the pre-and posttreatment values in Group 1, statistically significant differences were found across all parameters (*p* < 0.05). In Group 2, no statistical change was observed in the MOXO-CPT timeliness parameter (*p* > 0.05). However, significant improvements were observed in all other parameters (*p* < 0.05). In Group 3, no significant change was observed in the NHPT-nondominant parameter (*p* > 0.05), while improvements were seen in all other parameters (*p* < 0.05).

The intergroup comparisons of the pre-and posttreatment differences are shown in Table 2. Statistically significant differences were found in the parameters of the STS test, JPS-flex.Non-Dom., and JPS-abd.Non-Dom. (*p* < 0.05). No statistically significant differences were found between groups in the parameters of MOXO d-CPT attention, MOXO d-CPT timeliness, NHPT dominant, NHPT nondominant, JPS-flexion dominant, and JPS-abduction dominant. Group 1 was superior to Group 3 in the nondominant JPS-flexion, STS test, and nondominant JPS-abduction parameters (*p* < 0.017). When comparing Group 1 and Group 2, Group 1 was found to be more effective in the STS test parameter compared to Group 2 (*p* < 0.017). In contrast, the other parameters of the two groups were similar to one another (*p* > 0.017). No superiority was observed between Group 2 and Group 3 (*p* > 0.017).

## 4. Discussion

The current study investigated the effects of different video game–based therapy interventions combined with occupational therapy on cognitive functions, functional performance, proprioception, and fine motor skills in adults with mild to moderate ID.

The present study demonstrated that video game–based therapy programs, when integrated with occupational therapy interventions, positively impacted attention, functional performance, proprioception, and fine motor skills in individuals with ID. All intervention groups, combining both video game–based therapy and occupational therapy, were effective in improving attention, functional performance, proprioception, and fine motor skills at varying levels.

### 4.1. Cognitive Abilities

Attention and reaction time parameters were evaluated. Each intervention program was found to be effective for attention. There was no superiority between groups for attention. Cognitive abilities are an essential skill set that directly affects adaptive behavior and, subsequently, activities of daily living (ADL) among individuals with ID. Deficiencies in cognitive skills such as memory, sustained attention, and reaction time have an impact on procedural learning processes, and this leads to learning difficulties in ID [25]. The current study showed improvements in attention for all groups. Home-based supported intervention programs and occupational therapy–based activity training were found to be effective on reaction time, with higher significance in favor of home-based activity training. Various technology-based training methods in ID have been studied for cognitive abilities. Giachero et al. used action observation training through VR for procedural learning skills in adult individuals with ID. They suggested that only observation of activities positively affects attention and reaction time among the other tested cognitive parameters [26]. They utilized a VR setting to conduct action observation; thus, results were effective, similar to our study. In a scoping review, including studies using VR training for cognitive abilities, it was reported that VR-based training has promising benefits to enhance procedural learning for ADL [27]. In contrast to most studies, the present study utilized active video games rather than simulation of ADL in VR. Nevertheless, our study achieved similar effects on cognitive abilities.

### 4.2. Fine Motor Skills

In a study conducted on children and adolescents with physical disabilities, it was reported that Leap Motion–based video game exercises were effective in improving fine motor functions [28]. In the study conducted by Özkan and Kale, which examined the effectiveness of exercise training on quality of life and fine motor activities in individuals with IDs, it was reported that exercise training positively affected fine motor skills, upper extremity coordination, and quality of life in individuals with ID [29]. It has been indicated that a 14-week physical exercise and attention training program in children with moderate IDs improved visual retention, perception, attention, and motor skill levels [30]. In a meta-analysis study conducted by Zarei et al. on children with IDs, it was found that exercise training interventions improved both fine and gross motor skills in these children, highlighting the importance of regular exercise for individuals with ID [31]. Our study aligns with existing literature, as improvements in fine motor skills were observed in all three groups included in the study. It was considered that video game–based exercises and occupational therapy interventions specifically designed for the therapeutic goals improved fine motor skills in individuals with ID, and video game–based exercises could be an alternative treatment for enhancing fine motor skills.

### 4.3. Proprioception

Proprioception is essential to motor control and joint stability during daily activities. Impairments in proprioceptive awareness are common among individuals with ID. Proprioceptive awareness is a critical domain in the assessment of sensorimotor functioning. Disorders in proprioceptive awareness impact the precise organization of activities and affect reaction time [32]. Studies on video game–based therapy in adults with ID are limited. To our knowledge, no study in the literature evaluates joint position sense in individuals with ID. However, in a study investigating the effect of video-based game exercise interventions on shoulder proprioception, it was reported that active video-based game exercises improved the shoulder joint position sense [33]. Another study reported that strengthening exercises are effective in improving shoulder proprioception. It suggested that improvements in the sensitivity of muscle spindles and, hence, better neuromuscular control in the shoulder are achieved [34].

In our study, all intervention groups exhibited improvement in the JPS parameters. We believe this improvement is due to the selection of rehabilitative games explicitly designed for individuals' issues and used in rehabilitation settings, as opposed to exergames used for entertainment purposes. The task-oriented nature of these games and the execution of tasks at specific angles during gameplay contribute to the enhancement of joint position sense. Furthermore, we consider that the interventions applied facilitated neuromuscular control, thereby aiding in developing joint position sense. Additionally, our study observed that occupational therapy interventions in isolation improved JPS parameters. We hypothesize that the goal-oriented nature of the occupational therapy interventions we applied to individuals with ID may have contributed to the enhancement of JPS.

### 4.4. Functional Performance

Functional performance related to physical–motor skills among people with ID is an essential area since motor and physical problems can occur due to a lack of physical activity. With active video gaming, this physical activity level might increase with positive outcomes achieved for physical fitness. The present study shows that both video game–based therapy and occupational therapy–based activity have positive effects on functional performance. Results also indicate that home-based video game–based therapy may have superior effects on other groups or populations. This superiority might be due to the fact that such home-based video games are more inclusive, motivating, and challenging for participants. Studies using active video games for physical fitness in ID suggest positive effects of video game–based therapy. Wang et al. have reported that 8 weeks of video game–based therapy program in adolescents with ID improved lower extremity muscle strength. Similarly, they established a video game–based therapy setting using a Kinect sensor [35]. However, the present study differs from their study since different sensor systems have been used according to therapeutic aims.

A systematic review of randomized controlled trials included 13 studies investigating the effects of video game–based therapy on physical fitness (muscle strength, balance, coordination, etc.). Review results suggested that despite the various durations of treatment, systems used, and outcome measures utilized, video game–based therapy has positive effects on muscular fitness, balance, and cardiorespiratory fitness in individuals with ID [36]. Wiemeyer et al. emphasized the need for optimal exergame designs that require a multifaceted approach for individuals with disabilities. The researchers highlighted the necessity of developing an easy, enjoyable, and practical technology-based game program by considering children with developmental disorders' cognitive and physical characteristics [37]. In our study, games were played by the participants with the aim of improving their physical and cognitive skills. The ability to adjust the difficulty level of the games in our rehabilitation program according to the participants' conditions positively influenced their motivation. Cantone et al. reported that a visually focused, intensive occupational therapy program was more effective than traditional methods in improving daily living activities and motor skill parameters in individuals with mild IDs. Similarly, in our study, occupational therapy–based approaches and video-based rehabilitation interventions that incorporated visual and task-oriented feedback led to improvements in participants' physical performance and cognitive skills. The results of our study support these findings, suggesting that incorporating visual and task-oriented elements into rehabilitation interventions for individuals with IDs is crucial for achieving successful outcomes [38].

We believe that the present study holds a significant advantage in the literature, as it includes three active groups that were randomly assigned. Additionally, the use of home-based active video gaming and the implementation of different sensor systems tailored to specific therapeutic goals further strengthen the study's design.

## 5. Conclusions

In this study conducted on adults with IDs, it was observed that all three treatment methods were effective. Our results indicate that there were no significant differences in many parameters among the groups. We believe that including purposeful activities in occupational therapy interventions and video game–based exercise programs contributed to similar outcomes in evaluation parameters. We consider it essential to provide rehabilitative games as a home program for participants and to plan occupational therapy–based interventions to support this, as it holds significant value for individuals with ID. The limitations of this study included the duration of the treatment being set at 6 weeks and the absence of a longer-term treatment program, which is a notable limitation. Furthermore, in our study, improvements were observed in the participants' cognitive functions, functional performance, fine motor skills, and proprioception parameters. However, the impact of these improvements on individuals' quality of life has yet to be evaluated. The omission of quality of life assessment is considered a limitation in our research. Further comprehensive research is needed in this area.

For future research, we recommend exploring the use of occupational therapy and technology-based interventions across different disease groups, as well as evaluating the impact of technology-based occupational therapy on daily living activities, participation, and quality of life parameters. Additionally, assessing the long-term outcomes of such interventions is also suggested.

## Figures and Tables

**Table 1:** Comparison of the pretreatment and posttreatment values within the group.

**Variable**	**Pre-G1** **(** **m** **e** **a** **n** ± **SD)**	**Post-G1** **(** **m** **e** **a** **n** ± **SD)**	**p**	**Pre-G2** **(** **m** **e** **a** **n** ± **SD)**	**Post-G2** **(** **m** **e** **a** **n** ± **SD)**	**p**	**Pre-G3** **(** **m** **e** **a** **n** ± **SD)**	**Post-G3** **(** **m** **e** **a** **n** ± **SD)**	**p**
MOXO-CPT attention	9.67 ± 10.84	3.51 ± 4.83	< 0.01⁣^∗^	12.78 ± 12.21	6.49 ± 6.05	0.02⁣^∗^	12.11 ± 11.73	6.79 ± 7.81	0.01⁣^∗^
MOXO-CPT timeliness	3.87 ± 2.49.	0.22 ± 2.77	< 0.01⁣^∗^	4.38 ± 3.20	3.24 ± 1.94	0.24	5.17 ± 3.23	3.81 ± 2.60	0.04⁣^∗^
Sit-to-stand test	12.80 ± 3.85	19.00 ± 4.07	< 0.01⁣^∗^	11.73 ± 4.39	15.33 ± 3.79	< 0.01⁣^∗^	11.26 ± 2.52	14.80 ± 2.98	0.01⁣^∗^
NHPT-Dom.	30.02 ± 11.29	24.21 ± 8.97	< 0.01⁣^∗^	33.87 ± 24.63	26.42 ± 13.59	0.02⁣^∗^	38.92 ± 14.10	29.54 ± 10.55	0.01⁣^∗^
NHPT-Non-Dom.	29.08 ± 9.91	23.41 ± 7.85	< 0.01⁣^∗^	29.21 ± 10.31	24.91 ± 7.89	< 0.01⁣^∗^	42.48 ± 33.24	33.55 ± 16.24	0.07
JPS-flex.-Dom.	8.86 ± 4.59	0.26 ± 0.79	< 0.01⁣^∗^	6.60 ± 4.06	0.80 ± 1.14	< 0.01⁣^∗^	9.00 ± 5.86	3.53 ± 3.96	< 0.01⁣^∗^
JPS-flex.Non-Dom.	8.40 ± 5.74	0.20 ± 0.56	< 0.01⁣^∗^	6.26 ± 3.39	1.06 ± 1.57	< 0.01⁣^∗^	5.86 ± 4.88	2.46 ± 3.22	< 0.01⁣^∗^
JPS-abd.-Dom.	6.93 ± 6.55	0.13 ± 0.35	< 0.01⁣^∗^	7.93 ± 4.92	1.53 ± 1.64	< 0.01⁣^∗^	6.06 ± 5.52	3.06 ± 4.43	< 0.01⁣^∗^
JPS-abd.Non-Dom.	7.20 ± 3.83	0.53 ± 0.74	< 0.01⁣^∗^	5.73 ± 3.12	1.33 ± 1.39	< 0.01⁣^∗^	7.60 ± 7.78	4.26 ± 5.76	< 0.01⁣^∗^

Abbreviations: abd, abduction; CPT, continuous performance test; Dom, dominant; flex, flexion; G1, Group 1; G2, Group 2; G3, Group 3; JPS, joint position sense; NHPT, nine-hole peg test; SD, standard deviation.

⁣^∗^Significant value.

**Table 2:** Intergroup analysis of pre- and posttreatment differences.

**Variable**	**G1** **(** **m** **e** **a** **n** ± **SD)**	**G2** **(** **m** **e** **a** **n** ± **SD)**	**G3** **(** **m** **e** **a** **n** ± **SD)**	**Diff ** **p**	**p** ** G1–G2**	**ES Cohen's ** **d**	**p** ** G1–G3**	**ES Cohen's ** **d**	**p** ** G2–G3**	**ES Cohen's ** **d**
MOXO-CPT attention	6.15 ± 8.09	6.28 ± 9.95	5.32 ± 7.52	0.94	0.99	0.01	0.96	0.10	0.95	0.10
MOXO-CPT timeliness	3.64 ± 2.86	1.13 ± 3.60	1.35 ± 2.43	0.06	0.68	0.77	0.10	0.86	0.97	0.07
Sit-to-stand test	6.20 ± 1.85	3.60 ± 1.45	3.53 ± 1.84	< 0.01⁣^∗^	< 0.01⁣^∗^	1.56	< 0.01⁣^∗^	1.44	0.99	0.04
NHPT-Dom.	5.81 ± 3.71	7.45 ± 11.70	9.38 ± 5.73	0.46	0.83	0.18	0.43	0.73	0.77	0.20
NHPT-Non-Dom.	5.66 ± 3.88	4.29 ± 2.89	8.93 ± 18.08	0.48	0.93	0.43	0.68	0.25	0.47	0.35
JPS-flex.-Dom.	8.60 ± 4.38	5.80 ± 3.38	5.46 ± 4.51	0.08	0.16	0.71	0.10	0.70	0.97	0.08
JPS-flex.Non-Dom.	8.20 ± 5.68	5.20 ± 2.48	3.40 ± 4.46	0.01⁣^∗^	0.16	0.68	0.01⁣^∗^	0.93	0.50	0.49
JPS-abd.-Dom.	6.80 ± 6.66	6.40 ± 4.57	3.00 ± 3.31	0.08	0.97	0.07	0.11	0.72	0.16	0.85
JPS-abd.Non-Dom.	6.66 ± 3.77	4.40 ± 3.08	3.33 ± 3.61	0.01⁣^∗^	0.19	0.65	< 0.01⁣^∗^	0.90	0.68	0.31

Abbreviations: abd, abduction; CPT, continuous performance test; Dom, dominant; ES, effect size; flex, flexion; G1, Group 1; G2, Group 2; G3, Group 3; JPS, joint position sense; NHPT, nine-hole peg test; SD, standard deviation.

⁣^∗^Significant value. Bonferroni correction was applied for multiple comparisons, and *p* value < 0.017 was considered significant.

## Data Availability

The data that support the findings of this study are available from the corresponding author upon reasonable request.

## Supporting Information

Supporting Information Additional supporting information can be found online in the Supporting Information section. Description of intervention programs. Table S1. Description of mobile video games. Table S2. Description of supervised video game–based therapy program. Table S3. Description of occupational therapy program.
