# Supplementary material for: The Effect of a Video-Based Game Exercise Program on Motor Skills, Proprioception, and Cognitive Functions in Individuals With Intellectual Disabilities
Source: Occup Ther Int. 2025 Jan 30;2025:8410494. doi: 10.1155/oti/8410494 (PMC11824387; doi:10.1155/oti/8410494)
Supplement: Supporting Information — Additional supporting information can be found online in the Supporting Information section. Description of intervention programs. Table S1. Description of mobile video games. Table S2. Description of supervised video game–based therapy program. Table S3. Description of occupational therapy program. [file 8410494.f1.docx]

**Supplementary: Description of intervention programs**

**Table S1. Description of Mobile Video Games**

| **Description** |  |
| --- | --- |
| **Becure Gates**  An avatar shapes a body position and user need to replicate exact body position in given time limit. | 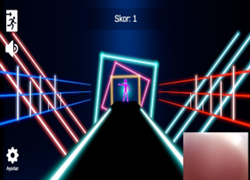 |
| **Becure Smart Clean**  User asked to clean the window in given time limit using upper extremity. | 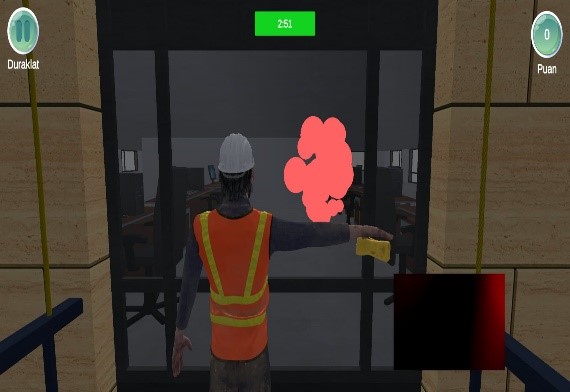 |
| **Becure Runner**  User controls the running avatar and tries to avoid obstacles by weight bearing laterally | 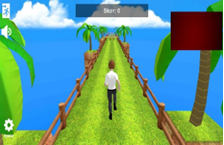 |
| **Becure Bubblepop**  User should use both upper extremities to pop the bubbles. Different colors popped by different extremity | 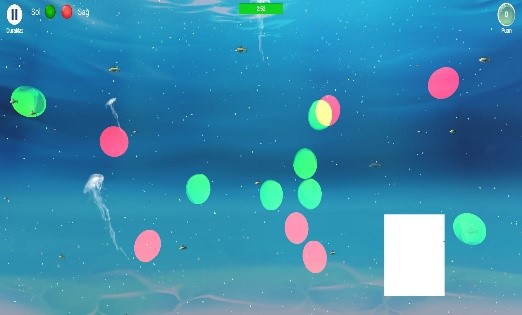 |

**Table S2. Description of supervised video game based therapy program**

| **Description** |  |
| --- | --- |
| **LeapBall**  User asked to grip the ball with hand and put the ball in holes | 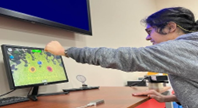 |
| **LeapPong**  It is a ping pong game which user controls the paddle with wrist flexion and extension. | 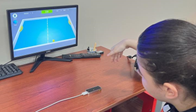 |
| **Kinect Balloon**  User requires to catch balloons with necessary degree of shoulder abduction. | 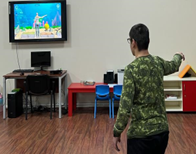 |
| **Kinect step on**  User use laterally stepping to catch balls. | 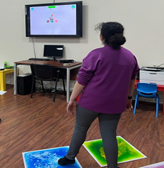 |
| **WBB Balance Surf**  User requires to weight bear laterally to control surf board. Tries to collect stars. | 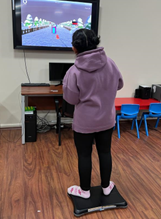 |
| **WBB Bowling**  User Requires to weight bear anter-posterior and laterally to control bowling ball. | 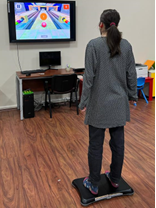 |

**Table S3. Description of Occupational Therapy Program**

| **Description** |  |
| --- | --- |
| While maintaining balance on a narrow surface, participant asked to remind the number given and touch the floor at the end | 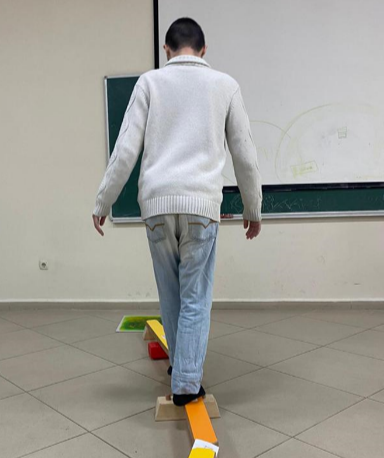 |
| Moving to the squares by jumping and placing balls | 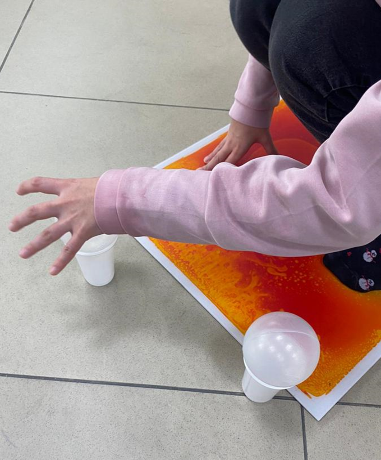 |
| Carrying ping pong balls with cup | 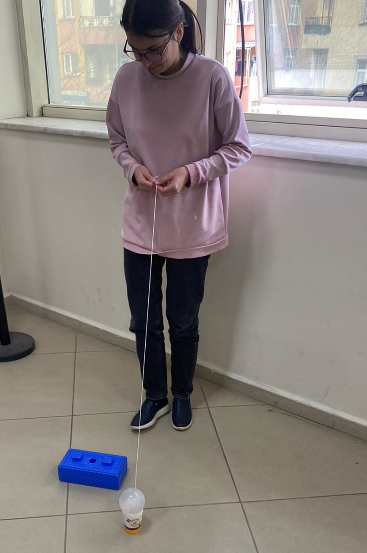 |
| Stringing beads activity | 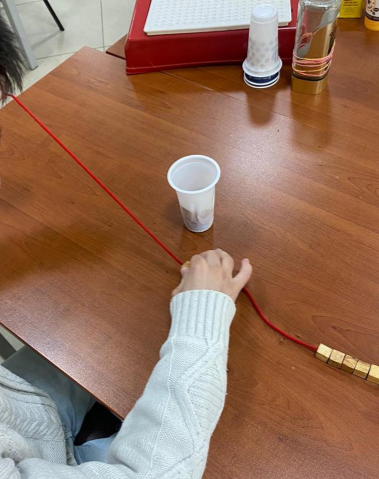 |
| Matching colors | 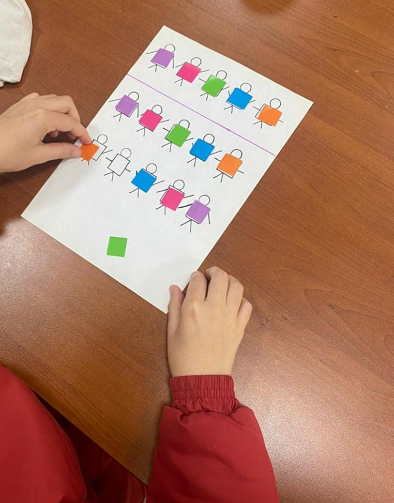 |
